# A Comprehensive Review of Antibiotic Resistance in the Oral Microbiota: Mechanisms, Drivers, and Emerging Therapeutic Strategies

**DOI:** 10.3390/antibiotics14080828

**Published:** 2025-08-15

**Authors:** Ena Kulis, Ivan Cvitkovic, Nikola Pavlovic, Marko Kumric, Doris Rusic, Josko Bozic

**Affiliations:** 1 Study of Dental Medicine, University of Split School of Medicine, 21000 Split, Croatia; kulis.ena@gmail.com; 2 Department of Anesthesiology and Intensive Care, University Hospital of Split, Spinciceva 1, 21000 Split, Croatia; ivcvitkovic@kbsplit.hr; 3 Department of Pathophysiology, University of Split School of Medicine, 21000 Split, Croatia; nikola.pavlovic@mefst.hr (N.P.); marko.kumric@mefst.hr (M.K.); 4 Laboratory for Cardiometabolic Research, University of Split School of Medicine, 21000 Split, Croatia; 5 Department of Pharmacy, University of Split School of Medicine, Soltanska 2, 21000 Split, Croatia; doris.rusic@mefst.hr

**Keywords:** oral microbiome, antimicrobial resistance, antimicrobial stewardship, oral AMR surveillance

## Abstract

Recent advances in microbiome research have highlighted the oral cavity as a complex and dynamic ecosystem, home to over 700 microbial species that play critical roles in both oral and systemic health. The oral microbiota not only maintains local tissue homeostasis but also serves as a reservoir for antimicrobial resistance (AMR) genes, contributing to the global spread of resistance. Frequent and sometimes inappropriate antibiotic use in dental practice, along with exposure to antiseptics and biocides, drives the emergence and horizontal transfer of resistance determinants within oral biofilms. This review synthesizes current knowledge on the molecular mechanisms and ecological drivers of AMR in the oral microbiome, emphasizing the clinical implications of dysbiosis and drug-resistant infections. The authors advocate for the development of dental clinical guidelines tailored to the unique characteristics of the oral microbiota, focusing on personalized therapy through molecular diagnostics, standardized AMR risk assessment, and the integration of non-antibiotic strategies such as probiotics and photodynamic therapy. Continuous education in antimicrobial stewardship and the implementation of oral-specific AMR surveillance is also highlighted as an essential component of effective resistance management. To support rational prescribing, a dedicated mobile application has been developed, leveraging microbiota data and resistance profiles to guide evidence-based, targeted therapy and reduce unnecessary antibiotic use. Collectively, these strategies aim to preserve antibiotic efficacy, ensure patient safety, and promote sustainable infection management in the dental field.

## 1. Introduction

### 1.1. The Oral Microbiome as a Microbial Niche

Until recently, the microbial communities inhabiting the human body were often referred to as “normal flora,” a term now considered outdated due to its vagueness and lack of functional context. More precise terminology has since emerged: “Microbiota” denotes the assemblage of microorganisms (bacteria, fungi, viruses, archaea) colonizing a specific niche, such as the oral cavity [1]. The “microbiome” extends this concept to include the collective genomes, metabolites, and interactions of these microbial populations with each other and the host environment. Finally, the “interactome” refers to the dynamic network of molecular and biochemical interactions among microbial communities and their host, shaping health and disease states. In this review, we adopt these definitions and use these terms consistently to enhance clarity [2,3].

### 1.2. Role in Health and Disease

The oral cavity represents a unique and complex microbial ecosystem, comprising over 700 microbial species, including bacteria, fungi, viruses, and archaea, which collectively contribute to both oral and systemic health. Unlike other body sites, the oral environment comprises diverse niches, including mucosal surfaces, dental hard tissues, and saliva, each of which supports distinct microbial communities adapted to specific ecological conditions. These microbial populations form structured biofilms, primarily dental plaque, which have an essential role in maintaining homeostasis but can also contribute to dysbiosis and disease when disturbed [4,5].

### 1.3. The Oral Cavity and Antimicrobial Resistance (AMR)

Importantly, the oral microbiome serves as a critical reservoir for antimicrobial resistance (AMR) genes, which can disseminate both locally and systemically. Frequent exposure to antibiotics through dental treatments, combined with the use of antiseptics and biocides in oral care products, creates selective pressure that facilitates the emergence and horizontal transfer of resistance determinants within oral biofilms [6,7,8,9]. This makes the oral cavity a hotspot for the exchange of resistance-conferring mobile genetic elements among diverse microbial species [10,11,12,13,14].

Consequently, understanding the molecular mechanisms driving AMR in this niche and the ecological factors influencing resistance development is vital. Such insights can guide improved clinical practices in dentistry, help tailor antimicrobial stewardship approaches, and inform public health strategies aimed at curbing the global rise in resistant infections [15,16].

Modern biomedical science increasingly utilizes comprehensive microbiome analyses [17,18], alongside investigations into the human virome [19,20,21,22,23], metagenome [17], and other omics layers such as the mycobiome, transcriptome, proteome, and metabolome [24,25,26,27,28,29,30]. These multi-omic approaches provide unprecedented insight into host–microbe interactions and the molecular underpinnings of health and disease. Accumulating evidence suggests that the microbiome and its collective functional output—the interactome—significantly influence most biological pathways affecting health, disease, and aging. Thus, the composition and activity of the microbiome are integral to virtually all biological processes throughout life [31]. Of particular concern is the role of the oral microbial community as a reservoir and vector for antimicrobial resistance (AMR). Due to constant exposure to antimicrobials, the oral cavity serves as a hotspot for the exchange of genetic material, or horizontal gene transfer, among microorganisms. Oral microbes can disseminate throughout the body and the environment, contributing to the global AMR threat [2,3].

### 1.4. Known Antibiotic Resistance Genes (ARGs) in the Oral Microbiome

The oral cavity harbors microorganisms carrying antibiotic resistance genes (ARGs) and mobile genetic elements that facilitate AMR dissemination. The *tetM* gene, often associated with the conjugative transposon Tn916, is the most prevalent [32]. *Enterococcus faecalis* exhibits significant tetracycline resistance, with the *tetM* gene detected in up to 60% of isolates. In contrast, the *ermB* gene mediates erythromycin resistance in approximately 61.9% of samples. Periodontal pathogens demonstrate resistance to beta-lactams, with *blaZ* and *cfxA* genes found in 90–97% of specimens. *Streptococcus salivarius*, a common oral commensal, carries the *mefA*/*E* gene, which confers erythromycin resistance in approximately 65% of cases [33]. A comprehensive study involving over 1400 subjects identified 159 ARGs conferring resistance to 22 antibiotic classes. The highest abundance of ARGs was observed in supragingival biofilms and saliva, with 49 ARGs distributed across various oral niches. Resistance to tetracyclines and macrolides was widespread, particularly in individuals with dental caries [33]. Some ARGs have been linked to severe infections such as infective endocarditis, and multidrug-resistant ESKAPE pathogens—clinically significant bacteria—have been detected in the oral microbiome [33].

### 1.5. Rationale and Objectives of the Review

Despite growing recognition of the oral microbiome’s complexity and its role as a reservoir of antimicrobial resistance (AMR) genes, a limited synthesis of factors driving AMR specifically within oral microbial communities remains. Previous reviews have often addressed AMR in broader human microbiomes or focused on systemic infections, leaving gaps in understanding oral-specific resistance genes, their mechanisms, and their direct clinical implications in dentistry. Moreover, the unique environmental pressures in the oral cavity, such as exposure to antiseptics, biocides, and frequent but sometimes inappropriate antibiotic use, create distinctive ecological and evolutionary conditions that foster the development of resistance, warranting dedicated investigation. This narrative review aims to fill this gap by comprehensively examining the molecular mechanisms and environmental drivers of AMR in the oral microbiota and discussing emerging therapeutic strategies. Given the increasing threat that drug-resistant oral pathogens pose to both local oral health and systemic disease, this timely synthesis is essential to inform clinical practice and guide future research and public health interventions. Of important note, evidence from neonatal microbiome research demonstrates that systemic antibiotic exposure—even outside the dental setting—can substantially alter oral microbial composition, underscoring the interconnectedness of antibiotic use and oral microbiota development from the earliest stages of life, highlighting the potential for long-term impacts on microbial ecology and the dissemination of antimicrobial resistance determinants [34].

**Figure 1:**
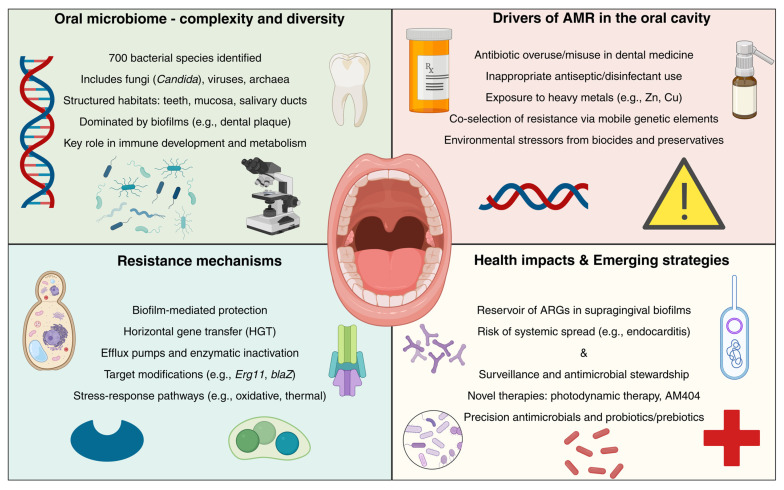
The oral cavity as a nexus of microbial diversity, antimicrobial resistance, and emerging therapeutic strategies. This figure highlights the oral microbiome as a central hub, illustrating its ecological structure, the external pressures that promote antimicrobial resistance (AMR), the molecular and cellular mechanisms underlying resistance, and the clinical implications that drive the development of targeted strategies to mitigate resistance and preserve oral and systemic health.

Despite extensive research on the human microbiome, significant knowledge gaps remain regarding the specific drivers and molecular mechanisms of antimicrobial resistance (AMR) within the oral microbiome. The current literature often lacks a comprehensive synthesis of the unique oral-specific resistance genes, their ecological dynamics, and clinical consequences for dental health. This review aims to fill these gaps by systematically examining the molecular mechanisms underlying AMR in oral microbial communities, the environmental and clinical factors that promote resistance emergence and dissemination, and the potential for emerging therapeutic strategies to combat resistant infections in the oral cavity. Given the oral cavity’s role as a reservoir and transmission hub for resistant pathogens and genes, understanding these processes is critical for developing targeted antimicrobial stewardship policies and improving public health outcomes. Thus, this narrative review provides a timely and focused synthesis that is essential for informing both dental clinical practice and future research priorities. The complex interplay between microbial diversity, environmental drivers, resistance mechanisms, and emerging interventions within the oral cavity is summarized in Figure 1.

## 2. Sources and Drivers of AMR in the Oral Cavity

### 2.1. Antibiotic Prescribing Practices in Dentistry

Antibiotics remain among the most frequently prescribed medications in dentistry; however, their use is sometimes unwarranted or inappropriate for the patients receiving them. Various and fluctuating factors influence the quality of prescription practices, underscoring the need for the development of targeted educational programs and workshops designed to enhance the competency of dental practitioners in rational drug prescribing [35].

Antimicrobial resistance within dentistry remains a growing concern, compounded not only by the misuse of antibiotics but also by the improper use of oral antiseptics. Antibiotic prescription should be reserved strictly for clinical scenarios that warrant empirical antimicrobial therapy and is not justified for all odontogenic infections. Systemic antibiotics, in particular, should be administered solely in specific cases that indicate their necessity. The two primary clinical indications for antibiotic use in dental practice are the prophylaxis of bacterial endocarditis and the prevention of surgical site infections [35].

### 2.2. Co-Selection of Antibiotic Resistance by Non-Antibiotic Agents

Recently, growing concern has emerged regarding the co-selection of antibiotic resistance among bacteria exposed to non-antibiotic antimicrobial agents, including biocides such as disinfectants, antiseptics, and preservatives, as well as heavy metals like copper and zinc [36]. Copper and zinc are widely recognized for their antimicrobial properties and are commonly incorporated into various oral healthcare products such as mouthwashes, toothpastes, and dental materials. Copper ions exert potent bactericidal effects by generating reactive oxygen species, disrupting bacterial membranes, and interfering with essential enzymatic processes, thereby reducing oral biofilm formation and microbial viability. Zinc ions similarly demonstrate broad-spectrum antimicrobial activity, inhibiting bacterial glycolytic enzymes, reducing plaque accumulation, and exhibiting anti-inflammatory properties that contribute to improved periodontal health [37].

Clinical formulations containing zinc, such as zinc citrate and zinc chloride, have been shown to effectively reduce halitosis and inhibit the growth of *Streptococcus mutans*, a primary cariogenic microorganism, thereby contributing to caries prevention. Moreover, copper-containing dental materials and coatings exhibit sustained antimicrobial effects, helping to prevent biofilm-associated infections on dental prostheses and implants [38].

However, the extensive use of these metals raises concerns about co-selection of resistance. Bacteria can harbor linked metal resistance genes (MRGs) and antibiotic resistance genes (ARGs) on mobile genetic elements, facilitating the development of co-resistance that may compromise antibiotic efficacy. Studies have detected plasmids simultaneously carrying copper resistance and multiple ARGs in oral and environmental bacterial isolates, underscoring the need for careful evaluation of copper and zinc use in oral health products to balance antimicrobial benefits with resistance risks [39,40].

Therefore, while copper and zinc represent valuable adjunctive agents in non-antibiotic antimicrobial strategies, their clinical application should be accompanied by continued surveillance of resistance development and integrated into comprehensive antimicrobial stewardship efforts within the dental field.

Both experimental and observational evidence suggest that exposure to these agents can induce or select for bacterial adaptations that reduce susceptibility to one or more antibiotics. Such resistance may arise through nonspecific protective mechanisms affecting multiple classes of antimicrobial compounds or via the selection of genetic resistance determinants physically linked to antibiotic resistance genes [40,41]. Furthermore, the impact of biocides and metals can manifest through alterations in microbial community structure, the activation of mobile genetic elements, and the induction of mutagenesis. Of particular concern is the observation that specific co-selective adaptations may negatively impact bacterial fitness and survival in the absence of selective pressure [41]. Antibacterial biocides and metals may drive co-selection of antibiotic resistance when bacteria harbor genes conferring resistance to both types of compounds—biocide/metal resistance genes (BMRGs) and antibiotic resistance genes (ARGs), which frequently co-occur. Despite numerous reported instances, systematic data on the co-occurrence of BMRGs and ARGs on plasmids and chromosomes, as well as their environmental and taxonomic distributions, remain scarce, complicating comprehensive risk assessment [40].

The outbreak of the SARS-CoV-2 pandemic has led to a marked increase in the use of disinfectants and antiseptics (DAs), resulting in elevated concentrations of these compounds in wastewater and receiving aquatic environments. The persistent presence of these agents may promote the development of microbial resistance to DAs and facilitate the emergence of cross-resistance to antibiotics. Compounds such as quaternary ammonium compounds (QACs), triclosan, and chlorhexidine have been particularly implicated in this phenomenon, in contrast to alcohol-based disinfectants [42].

## 3. Mechanisms of Resistance in Oral Microorganisms

### 3.1. Resistance in Candida and Other Oral Fungi

*Candida albicans* naturally forms biofilms, organized, three-dimensional multicellular structures that develop on various biotic and abiotic surfaces. These biofilms predominantly form within the host organism or on medical implants. Cells within these biofilms exhibit markedly reduced susceptibility to antifungal agents, which complicates eradication efforts. Concurrently, they demonstrate decreased vulnerability to immune system responses, which facilitates prolonged survival and contributes to the persistence of chronic infections. This combined resistance to antifungal therapy and immune evasion positions the biofilm as a central pathogenic factor in infections caused by this opportunistic fungus [43]. Fungal infections caused by *Candida* species represent a significant global health challenge due to high mortality rates and limited therapeutic options. *Candida albicans* remains the most common etiological agent of invasive candidiasis; however, the increasingly frequent isolation of resistant species, such as *Candida auris* and *Candida glabrata*, further complicates treatment strategies [44]. Beyond well-characterized resistance mechanisms, including decreased membrane permeability, enhanced efflux, and alterations in target molecules, alternative resistance pathways have been identified. These mechanisms encompass cellular stress responses to oxidative, thermal, and nutritional stresses, mediated by specific transcription factors such as TAC1 in *C. albicans* and YRR1 in yeast species.

Fungal pathogens such as *Candida albicans*, *Aspergillus fumigatus*, and *Cryptococcus neoformans* acquire resistance through intrinsic mechanisms, independent of prior drug exposure, as well as via resistance developed during prolonged antifungal therapy, often involving alterations in gene expression. Newly identified adaptive pathways to antifungal agents further complicate treatment and underscore the urgent need to elucidate the regulatory networks governing resistance [45].

Polyene resistance primarily arises from mutations that inactivate genes involved in ergosterol biosynthesis, resulting in decreased levels of the target sterol within the cellular membrane and consequently reducing drug efficacy [44]. In the case of azoles, resistance mechanisms include reduced sensitivity of the target enzyme (Erg11), its overexpression driven by regulatory mutations or aneuploidy, and elevated activity of efflux pumps that extrude the drug from the cell. The regulation of these efflux pumps is mediated by mutations in transcription factors, which vary among fungal species [44]. Echinocandin resistance is associated with mutations in genes encoding enzymes responsible for cell wall synthesis [40].

### 3.2. Resistance Mechanisms in Oral Bacteria

*Porphyromonas gingivalis* is a Gram-negative bacterium that plays a pivotal role in the pathogenesis of periodontal diseases and is frequently associated with multidrug-resistant (MDR) infections, posing significant therapeutic challenges [46]. Its pathogenicity is primarily attributed to the secretion of diverse virulence and antimicrobial factors that facilitate effective colonization, biofilm formation, and modulation of the host immune response. Although efflux pumps are well-established resistance mechanisms in numerous bacteria, their specific role in *Porphyromonas gingivalis* remains incompletely understood [46]. Chronic infections caused by this pathogen can lead not only to tooth loss but also contribute to the development of systemic diseases, underscoring the urgent need for novel therapeutic strategies. Within this context, four genes—*PG_0538*, *PG_0539*, *PG_0285*, and *PG_1797*—have been identified as potential targets for drug development. Notably, *PG_0539* and *PG_1797* have been shown to have associations with efflux pump function and virulence, opening new avenues for targeted therapies against MDR *P. gingivalis* strains [46].

Studies have confirmed the presence of antibiotic-resistant oral pathogens, including *Porphyromonas gingivalis*, *Prevotella* spp., *Fusobacterium nucleatum*, and *Aggregatibacter actinomycetemcomitans*, with notable resistance observed against amoxicillin, clindamycin, and metronidazole. Although overall resistance rates remain below 10%, regional variations complicate the establishment of universal treatment guidelines [47]. While resistance in patients with periodontitis has not yet reached critical levels, the emerging upward trend necessitates the implementation of strategies, including rapid diagnostics, rational antibiotic stewardship, and comprehensive education for healthcare professionals, to preserve the efficacy of antibiotics [47].

### 3.3. Novel Antimicrobial Strategies and Compounds

Standard treatment protocols for periodontal infections primarily involve mechanical debridement of the biofilm coupled with the administration of antiseptics or antibiotics. However, due to the increasing prevalence of antimicrobial resistance and the limited efficacy of conventional therapies, alternative treatment modalities are under investigation. In this context, the antibacterial and antibiofilm properties of AM404, a metabolite of paracetamol, have been evaluated. AM404 selectively inhibited both the growth and biofilm formation of *Porphyromonas gingivalis*, including biofilms formed on titanium surfaces, which is particularly relevant for the management of peri-implantitis. Its mechanism of action appears to depend on an unsaturated carbon chain and likely involves disruption of bacterial membrane permeability. Importantly, AM404 exhibited no cytotoxic effects on mammalian cells at concentrations up to four times the minimum inhibitory concentration (MIC), highlighting its therapeutic potential [48].

A longitudinal study spanning two decades in the United States revealed a dramatic increase in *Porphyromonas gingivalis* resistance to clindamycin and amoxicillin in patients with severe periodontitis, approximately 15-fold and 28-fold increases, respectively, compared to resistance levels recorded in 1999–2000. However, the study’s findings should be interpreted considering regional variations in antimicrobial use, the limited sample size for certain subgroups, and potential differences in methodology over time, which may influence the generalizability of these results. In contrast, susceptibility to metronidazole, its combination with amoxicillin, and doxycycline remained stable. Significant regional differences and the presence of diverse resistance genes underscore the necessity for localized surveillance programs and prudent antibiotic use to mitigate the spread of resistant strains and preserve therapeutic efficacy [49]. A summary of the major mechanisms, drivers, and emerging strategies for combating antibiotic resistance in the oral microbiota is provided in Table 1.

## 4. Clinical Implications

### 4.1. Prevalence and Identification of Antibiotic-Resistant Bacteria in the Oral Microflora of Children

A study conducted in Pakistan investigated the prevalence and characterization of antibiotic-resistant bacteria within the oral microbiota of healthy children, emphasizing the potential clinical implications for dental practice, particularly in the pediatric population. The research involved a healthy cohort of children aged 7 to 12, providing valuable insights into the prevalence and types of antibiotic-resistant bacteria present in the oral cavity. Notably, age- and sex-related differences were observed in resistance frequencies against various antibiotic classes [56].

In pediatric populations, antimicrobial resistance tends to increase with age, likely reflecting cumulative antibiotic exposure. For example, resistance to penicillins, cephalosporins, and macrolides was generally higher in the older subgroup (10–13 years) compared to the younger group (7–9 years), with penicillin resistance rising from approximately 18% to 23% across these age groups. Gender differences were also observed: males showed higher resistance rates to penicillins and macrolides, whereas females exhibited higher resistance to cephalosporins. Site-specific analysis identified the buccal mucosa as a common reservoir for bacteria that are resistant to antibiotics. At the species level, certain pathogens demonstrated markedly elevated resistance, with *Staphylococcus aureus* showing up to 50% resistance to penicillins and 33% to cephalosporins, while *Escherichia coli* and *Enterococcus faecalis* exhibited resistance levels often exceeding 30% for multiple antibiotic classes. These findings underscore the persistent presence of resistant bacteria even in younger cohorts, highlighting the need for vigilant antimicrobial stewardship in pediatric dental care [57].

Confounding factors, such as individual antibiotic exposure history, dietary habits, and environmental context (e.g., urban vs. rural living conditions), may influence these resistance patterns; hence, caution is advised when generalizing these results.

These findings have significant clinical relevance, informing antibiotic stewardship, particularly within preventive and therapeutic frameworks in pediatric dentistry. Nonetheless, the geographic specificity of the study population may limit the generalizability of these results, warranting cautious extrapolation to other demographic contexts without further corroborative research.

### 4.2. Diversity of Bacterial Communities and Prevalence of Antibiotic Resistance Genes in the Oral Microbiota

Almeida et al. conducted a study in Brazil analyzing the diversity of bacterial communities and the prevalence of antibiotic resistance genes (ARGs) in the oral microbiota. Their findings provided insights into the relationship between periodontal health status and the presence of resistance genes, which may impact treatment outcomes. Their investigation compared microbial diversity and ARG prevalence between individuals with healthy periodontium and those with periodontal disease [58].

The study included 110 participants, from whom saliva and subgingival plaque samples were collected and analyzed via 16S rRNA gene sequencing to assess bacterial diversity. Assessment of periodontal health revealed that while nearly a quarter of participants had healthy periodontal conditions, the majority were affected by gingivitis (55.4%) or chronic periodontitis (21.8%). Microbial profiling showed distinct shifts in community composition between healthy and diseased states, with increased predominance of Streptococcus and Rothia genera in diseased samples.

Resistance gene screening demonstrated that over 70% of samples harbored at least one antibiotic resistance gene (ARG), with macrolide resistance genes (erm) being the most common, identified in 58.2% of cases. Beta-lactamase genes such as blaTEM were detected less frequently (16.4%), while resistance determinants like mecA, pbp2b, and aac (6′) occurred at lower prevalence (around 6%). These data suggest a widespread reservoir of resistance within oral microbial communities, which may complicate treatment outcomes in periodontal disease [58].

It is important to consider that factors including prior antibiotic use, oral hygiene practices, and demographic variables could affect ARG prevalence and distribution, emphasizing the complexity underlying resistance epidemiology in the oral cavity [57].

Although no significant differences were observed in the overall taxonomic structure of the microbiome between healthy and diseased groups, samples from healthy individuals exhibited greater bacterial diversity, suggesting a more stable and resilient microbial community. The high prevalence of ARGs, particularly those related to antibiotics frequently employed in dentistry, underscores the critical need for prudent and rational antimicrobial prescribing practices. Moreover, ongoing surveillance of resistance within the oral environment is essential to prevent the dissemination of resistance determinants to systemic sites [57].

### 4.3. Dental Plaque Microbial Resistomes in Periodontal Health and Disease

This study employed metagenomic sequencing to analyze dental plaque and investigate resistance gene profiles in patients before and after scaling and root planing (SRP) therapy. A significant shift in the resistance gene repertoire was observed post-therapy, with important clinical implications for antibiotic selection and therapeutic strategies.

A cross-sectional study analyzing dental plaque samples from three groups—healthy individuals, patients with periodontitis before treatment, and patients post-scaling and root planing (SRP)—reported that periodontitis was associated with an increased abundance and altered composition of antibiotic resistance genes (ARGs) and metal resistance genes (MRGs) compared to healthy controls. Notably, SRP treatment further influenced the oral resistome: certain ARGs and MRGs increased in abundance following therapy, suggesting that mechanical biofilm disruption and subsequent microbial shifts may transiently modify resistance gene profiles. However, the clinical relevance of these findings remains unclear. The observed post-treatment increases may reflect short-term ecological perturbations rather than sustained antimicrobial resistance. Larger, longitudinal studies are needed to determine the long-term effects of SRP on the oral resistome and its potential implications for resistance management [58].

The dominant ARG classes identified included genes conferring resistance to bacitracin, beta-lactams, macrolide–lincosamide–streptogramin (MLS) antibiotics, tetracyclines, and multidrug resistance. Regarding MRGs, genes associated with resistance to multiple metals, such as iron, chromium, and copper, were predominant [53]. Key bacterial species linked to disease progression, including *Filifactor fastidiosum*, *Tannerella forsythia*, and *Campylobacter rectus*, were identified. The detection of mobile genetic elements suggests the co-selection of resistance traits [58].

These results elucidate the intricate interactions among microbial communities, resistance genes, and genetic mobility within the oral microbiome, underscoring the complexity of antimicrobial resistance dynamics in periodontal health and disease.

### 4.4. Oral Health Management in the Context of Antimicrobial Resistance

#### 4.4.1. Education and Stewardship

This review article highlights recent advances in the understanding of the oral microbiome and resistome, providing an updated overview of how dental practitioners are adapting to the treatment of oral infections in light of the antimicrobial resistance (AMR) challenge [59].

The oral microbiome plays a pivotal role in shaping oral health. Consequently, the preservation and restoration of a balanced oral microbiome represents a primary objective, as well as a significant challenge for dental professionals. The colonization of pathogenic microorganisms within the oral cavity and their integration into biofilms frequently precipitate the development of gingivitis, caries, periodontitis, peri-implantitis, and other oral infections. For instance, preclinical evidence demonstrates that long-term systemic antibiotic exposure can induce gut dysbiosis, which in turn disrupts the oral microbiota, exacerbates periodontitis, and promotes alveolar bone loss through a Th17/Treg immune imbalance. These findings underscore the potential for systemic antibiotic use—even when unrelated to dental care—to negatively influence oral health [60]. Currently, several therapeutic and prophylactic modalities are available, the majority of which are antibiotic-based. Given the rising threat of antimicrobial resistance, the prudent regulation and rational use of antibiotics in dentistry constitutes an essential strategy to optimize clinical outcomes and prevent unnecessary and excessive antibiotic administration, an integral step toward precision medicine. Further progress depends on the development of novel and effective treatment modalities to supplant antibiotics, including photodynamic therapy and the application of probiotics [59].

Dental indications for antibiotic administration primarily fall into two categories: firstly, to improve surgical outcomes and reduce symptoms and complications, and secondly, for the treatment of infections [60]. Traditionally, the indication for antibiotic use in dental practice has been based largely on clinical experience, subjective assessment, or outdated evidence rather than on effective diagnostics, resulting predominantly in empirical administration with an emphasis on broad-spectrum antibiotics [60,61,62]. Guidelines for rational antimicrobial prescribing were rarely disseminated among dental practitioners and remained scarce [63]. Furthermore, recommendations for prudent antibiotic use vary geographically, with different approaches being advised based on the resistance patterns specific to individual countries. Moreover, these guidelines lack international consensus and often diverge [64,65,66,67,68,69,70]. Taken together, these observations underscore the critical need for implementing antibiotic stewardship programs within dentistry [71]. Effective management of rational antibiotic use necessitates a coordinated set of interventions aimed at optimizing the selection, dosage, duration, and route of administration to enhance clinical outcomes, minimize adverse effects, and reduce the emergence of resistance [72]. The multidisciplinary antibiotic stewardship team should ideally comprise dentists, pharmacists, microbiologists, and other healthcare professionals [73].

Effective antibiotic stewardship must adopt coordinated interventions that optimize antibiotic selection, dosage, duration, and administration route to maximize efficacy, minimize side effects, and reduce the emergence of resistance. Multidisciplinary stewardship teams, ideally including dentists, pharmacists, microbiologists, and other healthcare professionals, can enhance rational antibiotic use. Key measures include continuous education for dental teams and patients on AMR risks and rational antibiotic use, alongside targeted training for dental students emphasizing evidence-based prescribing [74,75,76,77,78]. Educational programs incorporating workshops, lectures, practical exercises, and online courses have demonstrated positive impacts on prescriber knowledge and behavior. Harmonizing stewardship protocols internationally is essential to facilitate consistent antibiotic use comparisons and improve care standards globally [79].

#### 4.4.2. Guidelines and Practices

Antibiotic therapy has demonstrated efficacy as an adjunctive treatment for periodontitis; however, its rational use remains imperative due to the increasing prevalence of antimicrobial resistance. Notably, resistance incidence is significant among species such as *Porphyromonas gingivalis*, *Prevotella intermedia*, *Prevotella denticola*, *Prevotella melaninogenica*, *Fusobacterium nucleatum*, *Tannerella forsythia*, *Aggregatibacter actinomycetemcomitans*, *Streptococcus constellatus*, *Streptococcus intermedius*, and *Parvimonas micra* [80]. Although resistance rates have generally remained below 10% in most studies, exceptions include *Aggregatibacter actinomycetemcomitans* resistance to amoxicillin. The highest prevalence of resistance across bacterial species was observed for amoxicillin, clindamycin, and metronidazole. However, resistance patterns showed considerable geographic variability [80].

In patients with peri-implantitis, *Porphyromonas gingivalis* and *Fusobacterium nucleatum* exhibited high levels of resistance to tetracycline, metronidazole, and erythromycin. Similarly, *Aggregatibacter actinomycetemcomitans* demonstrated considerable resistance to clindamycin and doxycycline. *Tannerella forsythia*, *Parvimonas micra*, and *Prevotella intermedia*/*nigrescens* showed significant resistance to other antibiotics, including azithromycin, moxifloxacin, and amoxicillin. However, resistance was generally not observed among most microorganisms when amoxicillin was combined with metronidazole [81]. The use of dual or adjunctive antibiotic therapy in these cases remains controversial; nevertheless, this review found such combination therapies to be effective, contrasting with classical monotherapy regimens. When prescribing antibiotics, clinicians should carefully consider resistance patterns, targeted therapy approaches, and appropriate alternatives and combinations of antibiotics [81].

*Aggregatibacter actinomycetemcomitans*, *Tannerella forsythia*, and *Porphyromonas gingivalis* exhibit low resistance to amoxicillin; however, these microorganisms demonstrate resistance to tetracycline, metronidazole, and azithromycin at various sites before treatment [82]. *A. actinomycetemcomitans* showed high resistance to tetracycline both before and after therapy [82]. The local administration of antibiotics, either alone or as an adjunct to surgical or non-surgical interventions in peri-implantitis management, has demonstrated positive outcomes, albeit with limited evidence. In contrast, systemic application alongside these procedures remains questionable [83]. The heterogeneity of antibiotics used, routes of administration, and treatment protocols restricts the ability to draw definitive conclusions regarding the most effective therapeutic strategies for peri-implantitis [83].

#### 4.4.3. Adjunctive Therapies

Dentists account for approximately 10% of all antibiotic prescriptions, with antimicrobial resistance predominantly linked to their irrational prescribing and usage practices. In response to this issue, techniques aimed at reducing or avoiding antimicrobial therapy as an adjunct to surgical and non-surgical interventions have been introduced, such as photodynamic therapy. The study by Zhao et al. reports promising results with this approach, although further research is necessary to substantiate these findings [84]. Other promising alternatives include probiotics, which may help restore microbial balance by promoting beneficial bacteria, and antimicrobial peptides that target pathogens selectively without disturbing commensals, thus preserving microbial homeostasis. These novel adjunctive approaches are expected to complement traditional therapy and aid in reducing the development and spread of AMR.

## 5. Alternative and Adjunctive Therapies

### 5.1. Use of Antimicrobial Peptides, Nanoparticles, and Photodynamic Therapy

Due to the growing challenge of antimicrobial resistance to antibiotics, novel therapeutic approaches have been developed, among which antimicrobial photodynamic therapy (aPDT) has gained prominence. aPDT employs photosensitizers that are activated by visible light to generate reactive oxygen species (ROS), which inflict damage on microbial cells and the biofilm matrix. Numerous studies have confirmed the efficacy of aPDT in reducing and eradicating biofilms [79]. Antimicrobial photodynamic inactivation (aPDI) is increasingly recognized as an effective strategy for biofilm removal, particularly in the treatment of chronic and recurrent infections. Its multifaceted mechanism involves ROS generated during treatment that not only kill microbial cells but also degrade the extracellular polymeric substance (EPS), thereby destabilizing the biofilm’s structural integrity [85,86].

In vitro investigations demonstrate significant efficacy of aPDI against Gram-negative bacteria such as *Pseudomonas aeruginosa* and *Aggregatibacter actinomycetemcomitans*, achieving bacterial load reductions of up to 10^7^-fold. The mode of action includes initial targeting of polysaccharides within the biofilm matrix, followed by membrane disruption and intracellular penetration. Comparable effectiveness has been observed against other species, including *Escherichia coli*, *Fusobacterium nucleatum*, and *Moraxella catarrhalis*, accompanied by notable morphological alterations [85,87]. In the context of fungal biofilms, particularly those formed by *Candida albicans* and *Candida parapsilosis*, aPDT has been demonstrated to result in the complete eradication of biofilms. The underlying mechanisms involve inhibition of cellular metabolism, damage to the cytoplasmic membrane, and increased cellular permeability. Photosensitizers such as methylene blue and toluidine blue have demonstrated high therapeutic efficiency [79]. Mixed-species biofilms, for instance, those comprising *Streptococcus mutans* in combination with *Lactobacillus acidophilus*, have been effectively disrupted using curcumin. Moreover, functionalizing photosensitizers with nanoparticles enhances their affinity for the cellular membrane, facilitating deeper penetration of biofilms and thereby augmenting therapeutic outcomes [85,88].

A systematic review and meta-analysis of 14 studies reported that antimicrobial photodynamic therapy (aPDT) utilizing photosensitizers (PS) conjugated with inorganic nanoparticles demonstrates enhanced efficacy without promoting antimicrobial resistance [83]. The most frequently studied microorganisms included *Staphylococcus aureus*, *Escherichia coli*, *Pseudomonas aeruginosa*, *Staphylococcus epidermidis*, *Enterococcus faecalis*, *Aggregatibacter actinomycetemcomitans*, *Porphyromonas gingivalis*, *Prevotella intermedia*, and *Candida albicans*. Most microorganisms were cultured in suspension form, while only five studies evaluated microorganisms within biofilms. Regardless of the type of inorganic nanoparticles or microbial species, all included studies reported a significant reduction in microbial load. Specifically, photosensitizers conjugated with inorganic nanoparticles have been shown to inhibit biofilm formation [47] significantly. The nanoparticles employed included metallic and silicon-based particles as delivery systems for the photosensitizers. Results indicated a marked reduction in microbial counts, especially for *E. coli* (OR = 0.08) and Gram-negative bacteria (OR = 0.12) [47]. Despite these promising outcomes, the studies exhibited biases related to blinding, underscoring the need for further research using biofilm models. Pathogenic microorganisms have evolved mechanisms of persistence, tolerance, and resistance, culminating in the emergence of multidrug-resistant strains that pose a significant medical challenge [47].

In a related systematic review and meta-analysis, Dias et al. investigated the combination of aPDT with antimicrobial peptides (AMPs) as an alternative strategy to address the growing issue of antimicrobial resistance [89]. AMPs are oligopeptides composed of up to 50 amino acids, produced by virtually all living organisms as components of innate immune defenses against pathogens. Characterized by a net positive charge, primarily due to arginine and lysine residues, and a high proportion of hydrophobic amino acids, AMPs effectively target negatively charged microbial membranes. Their activity is structure-dependent, typically adopting α-helical or β-sheet conformations. Most AMPs exert their antimicrobial effects via direct membrane disruption, although some target intracellular components [90,91]. Analysis of 20 in vitro studies demonstrated that the combination of aPDT and AMPs significantly reduced microbial burden (OR = 0.14), surpassing the efficacy of aPDT alone. However, only 20% of the studies assessed effects on biofilms, highlighting the need for further blinded investigations in biofilm models [90].

The study by de Freitas et al. (2018) [92] investigated the combination of antimicrobial photodynamic therapy (aPDT) and the antimicrobial peptide aurein 1.2 against *Enterococcus faecalis*. The results demonstrated that this combined treatment, particularly when using methylene blue or chlorin-e6 as photosensitizers (PS), could completely eradicate vancomycin-resistant *E. faecalis* and *E. faecium* at low concentrations of both PS and AMP [91]. The efficacy was dependent on the specific PS employed, with the combination representing a promising alternative to conventional antibiotics for managing localized infections [93].

However, despite the encouraging in vitro and preliminary clinical results supporting the antimicrobial efficacy of photodynamic therapy (aPDT), current clinical practice guidelines, including those published by the European Federation of Periodontology [94], recommend cautious applications. These guidelines note that while aPDT may serve as a promising adjunctive treatment, there remains insufficient high-quality clinical evidence from standardized, randomized controlled trials to endorse its routine use in periodontal and peri-implant therapy. The heterogeneity of study protocols, photosensitizers, and light sources, as well as limited long-term outcome data, precludes definitive conclusions on clinical efficacy. Consequently, aPDT should presently be considered an experimental or adjunctive option pending further robust clinical validation, and clinicians are advised to follow established evidence-based guidelines in their therapeutic decisions [94].

### 5.2. Role of Narrow-Spectrum Antibiotics and Targeted Delivery Systems

The authors developed positively charged polymeric nanoparticles with a glucosylated surface that enables specific absorption of antibiotics in the proximal small intestine via the sodium-dependent glucose transporter (SGLT1). This approach enhances the bioavailability of antibiotics while limiting their exposure to the colonic microbiota, thereby reducing the risk of intestinal dysbiosis [93]. In murine models, oral administration of ampicillin, chloramphenicol, or vancomycin encapsulated within these nanoparticles effectively eradicated pulmonary infections, mitigated detrimental effects on the gut microbiota, protected the animals from dysbiosis-associated metabolic syndromes, and decreased the accumulation of antibiotic resistance genes in commensal bacteria [93].

Additionally, the authors established a microencapsulation technique for *Escherichia coli* bacteriocins, colicins E9 and Ia, within pH-sensitive hydrogel microcapsules. This method facilitates the controlled release of bacteriocins in the lower gastrointestinal tract, minimizing their interaction with the gut microbiota and thereby reducing the potential for the development of antibiotic resistance [84]. Murine studies have demonstrated that the oral administration of encapsulated colicins significantly decreases intestinal colonization and fecal shedding of *E. coli* in animals [84]. This work highlights the potential of bacteriocins as narrow-spectrum antibiotic proteins for therapeutic modulation of the human gut microbiota. The developed targeted delivery system enables the precise administration of therapeutic proteins to specific regions of the gastrointestinal tract, minimizing adverse impacts on the healthy microbiota and reducing the risk of antibiotic resistance emergence.

Although local antibiotic applications can reduce systemic exposure and potential side effects, concerns remain that excessive or improper use of topical antibiotics may contribute to the development of localized antibiotic resistance [95]. Nanosystems have emerged as a promising solution. Nanocarriers such as liposomes, dendrimers, and polymeric nanostructures are being investigated for their ability to enhance antibiotic efficacy and potentially overcome bacterial resistance mechanisms. Further research is required to better understand the mechanisms of antibiotic resistance development in the context of local antibiotic use in periodontal disease treatment and to develop strategies minimizing this risk [95].

Various systems for targeted local antibiotic delivery into periodontal pockets have been developed, including microspheres, hydrogels, and liposomes. These systems enable controlled and sustained drug release, improving therapeutic efficacy and reducing the need for frequent administration. Microspheres, often composed of biocompatible materials such as poly(lactic-co-glycolic acid) (PLGA), enable the gradual release of minocycline hydrochloride, thereby decreasing the administration frequency and side effects. Similarly, liposomes can enhance drug stability and target specific sites within the oral cavity [89].

Experimental models have demonstrated that these delivery systems improve clinical outcomes by reducing periodontal pocket depth, decreasing pathogenic microbial load, and stimulating alveolar bone regeneration. These findings suggest that narrow-spectrum antibiotics combined with advanced drug delivery systems offer promising approaches for managing periodontal disease, minimizing negative impacts on the oral microbiota, and reducing the potential for antibiotic resistance development. This review highlights advances in oral nanoantibiotics development, including lipid-based nanoantibiotics, such as SNEDDS, liposomes, and solid lipid nanoparticles (SLNs), for antibiotic delivery in the oral cavity. The advantages of these systems include increased bioavailability and reduced side effects [92].

### 5.3. Probiotics, Prebiotics, and Microbiota Restoration Strategies

Recent decade-long research highlights the critical role of probiotics, prebiotics, and microbiota restoration strategies in maintaining oral microbial homeostasis and addressing the growing challenge of antibiotic resistance. These therapeutic modalities offer promising alternatives or adjuncts to conventional antibiotic regimens by mitigating dysbiosis and curtailing the emergence of resistant strains.

Specific probiotic taxa, notably *Lactobacillus* and *Bifidobacterium* species, have demonstrated potent inhibitory effects against pathogenic oral microorganisms. For instance, *Lactobacillus fermentum* ALAL020 exerts antimicrobial activity against *Porphyromonas gingivalis* through the biosynthesis of cyclic dipeptides, whereas *Lactobacillus acidophilus* La5 modulates local immune responses by downregulating pro-inflammatory cytokines. The mechanisms underlying probiotic efficacy encompass secretion of antimicrobial metabolites (e.g., cyclic dipeptides), competitive exclusion for nutrients and adhesion sites, acidification and disruption of pathogenic biofilms, immunomodulatory effects characterized by suppression of pro-inflammatory cytokines (IL-1β, IL-8) and enhancement of anti-inflammatory mediators (IL-10, TGF-β), as well as stimulation of host defense via activation of β-defensins and T-cell responses [96].

In the context of periodontitis management, probiotics emerge as valuable adjunctive agents, particularly given the rising prevalence of antibiotic resistance. Unlike broad-spectrum antibiotics, probiotics selectively target pathogenic species without perturbing commensal flora, thereby reducing the risk of dysbiosis. Clinical applications include probiotic-enriched dentifrices and mouth rinses, which have shown promising anti-inflammatory and anti-biofilm effects. However, therapeutic benefits are predominantly short-term, with evidence indicating a plateau in efficacy beyond one month of administration, highlighting the necessity for further longitudinal studies to ascertain sustained outcomes [91]. Probiotic strains, such as *Lactobacillus reuteri*, *L. fermentum*, *L. gasseri*, and *Streptococcus salivarius*, have consistently demonstrated efficacy in suppressing key periodontal pathogens (e.g., *Porphyromonas gingivalis*, *Aggregatibacter actinomycetemcomitans*), downregulating inflammatory cytokines (IL-1β, TNF-α, IL-17), modulating immune responses, and maintaining microbial balance. Meta-analytical data corroborate these findings; nonetheless, precise parameters regarding optimal treatment duration and safety profiles, especially among immunocompromised populations, remain to be fully elucidated [96].

Prebiotics, such as fructooligosaccharides (FOS) and galactooligosaccharides (GOS), selectively stimulate the growth of beneficial bacteria, while synbiotics, which are combinations of probiotics and prebiotics, exert synergistic effects. These compounds have been shown to attenuate inflammation, inhibit pathogenic microorganisms, and enhance microbial homeostasis within the oral cavity [97].

Halitosis is predominantly caused by biofilm formation and oral inflammation, with bacteria producing volatile sulfur compounds (VSCs) such as hydrogen sulfide (H_2_S) and methyl mercaptan (CH_3_SH) as primary contributors. Conventional treatments often provide only transient relief, as pathogenic species rapidly recolonize the oral environment. Probiotics facilitate halitosis reduction by promoting a healthy tongue microbiota and inhibiting VSC production. Specific strains, including *Streptococcus salivarius* K12, have demonstrated sustained efficacy following lozenge administration; *Weissella cibaria* has been shown to reduce H_2_S and CH_3_SH levels in pediatric populations, while *Lactobacillus salivarius* WB21 and *Lactobacillus reuteri* have effectively decreased malodor in chewing gum-based interventions [96].

**Figure 2:**
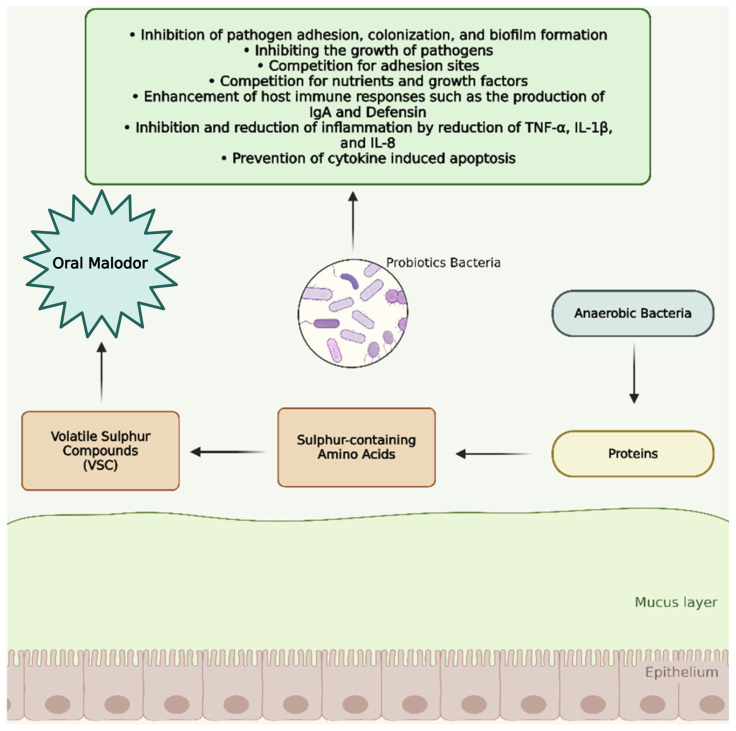
Role of probiotics in reducing oral malodor through sulfur compound pathway modulation. This Figure illustrates the role of probiotics in reducing oral malodor by inhibiting the activity of anaerobic bacteria responsible for the production of volatile sulfur compounds from sulfur-containing amino acids.

However, not all probiotic strains yield beneficial outcomes: for instance, *Lactobacillus brevis* CD2 and *Lactobacillus casei Shirota* did not exhibit significant effects on halitosis. Further rigorous research is warranted to evaluate commercially available probiotic strains and optimize their clinical applications in halitosis management [96]. The relationship between protein degradation, volatile sulfur compound production, and the modulatory effects of probiotics on oral malodor is summarized in Figure 2.

This paper reviewed seven studies (eight reports) involving individuals who received systemic antibiotics alongside probiotics during or after antibiotic therapy [96]. The findings were as follows: One study reported that probiotics mitigated changes in microbiome diversity, while another indicated that probiotics exacerbated these changes. Four studies found no significant effect, and one study documented an increase in antibiotic resistance genes. The overall conclusion is that the evidence is inconsistent and insufficient to definitively confirm the efficacy of probiotics in restoring the microbiome following antibiotic treatment.

## 6. Future Directions and Research Gaps

### 6.1. Need for Oral-Specific AMR Surveillance

The oral microbiota represents a significant reservoir of antimicrobial resistance (AMR) genes, yet it remains underexplored. Studies have identified a broad diversity of antibiotic resistance genes (ARGs) spanning numerous antibiotic classes, including tetracyclines, macrolides, and beta-lactams. One comprehensive investigation detected 64 ARGs covering 36 antibiotic classes across various clinical conditions but noted a discrepancy between genotypic data and phenotypic resistance, indicating that the presence of ARGs does not always equate to expressed resistance. Interestingly, healthy individuals and those with active dental caries harbored a greater abundance of ARGs compared to patients with periodontitis, a finding that may reflect differences in microbial ecology or methodological variability. The authors emphasized the need for standardized approaches that integrate both genotypic and phenotypic methods to reliably monitor oral AMR, ideally within broader public health surveillance systems [97,98].

Further supporting these observations, a study involving 110 adults, including healthy subjects and periodontitis patients, found ARGs present in nearly three-quarters of oral samples from saliva and subgingival plaque. Macrolide resistance genes such as *erm* were most prevalent, found in over half of the samples, followed by beta-lactamase genes like *blaTEM*, although resistance genes for critical drugs such as carbapenems or metronidazole were notably absent, which bears clinical relevance. The use of PCR-based molecular methods to detect ARGs from oral samples proved to be a practical and minimally invasive surveillance tool suitable for routine clinical applications. This highlights the oral cavity’s role as a dynamic reservoir capable of disseminating resistance genes to other microbiota within the host or environment, underscoring the need to include the oral niche in AMR monitoring programs [99].

Metagenomic analyses in patients with periodontitis shed further light on the complexity of oral AMR. Shotgun sequencing of subgingival pockets revealed nineteen distinct ARGs across six antibiotic classes commonly used in periodontal treatment, with macrolide resistance predominating. These findings underscore subgingival pockets as highly selective environments where resistance is likely driven and maintained. The ability of metagenomics to detect ARGs rapidly and without the need for culturing offers a valuable readout for future clinical decision-making. Importantly, these data support the movement towards personalized therapeutic strategies that may involve substituting systemic antibiotics with local therapies or adjunctive treatments such as photodynamic therapy to mitigate resistance development.

Despite these advances, several limitations temper the interpretation of oral AMR research. The variability in sample sizes, subject populations, and applied molecular techniques complicates the amalgamation of findings and limits generalizability. Insufficient correlation between genotype and phenotype suggests that exclusive reliance on either molecular or culture-based approaches cannot fully capture the functional resistance status. Moreover, geographic variation, environmental exposures, prior antibiotic use, dietary habits, and other host factors likely influence ARG prevalence and expression but are not yet systematically accounted for, indicating a need for more comprehensive, multifactorial studies [99].

Overall, there is consensus in the literature that standardized and oral-specific AMR surveillance protocols integrating genotypic and phenotypic assessments are urgently needed. Inclusion of the oral cavity within broader public health frameworks will be critical, especially given the frequent antibiotic prescribing practices in dental care that can select for resistance. Emerging molecular techniques, non-invasive sampling methods, and bioinformatics tools promise to revolutionize this surveillance, enabling more targeted and effective stewardship interventions. These steps are essential to reduce the clinical and public health burdens of antimicrobial resistance linked to the oral microbiome.

### 6.2. Precision Approaches to Microbiota Modulation

C16G2 is an innovative, targeted antimicrobial peptide specifically developed to combat *Streptococcus mutans*, the primary bacterial agent responsible for dental caries. Unlike broad-spectrum antibiotics that disrupt the overall oral microbiota, C16G2 exhibits high specificity for *S. mutans*, binding to surface molecules and destabilizing its cell membrane, leading to selective bacterial lysis without harming beneficial commensals. This specificity minimizes disruption of the oral ecosystem and reduces the potential for resistance development, a common limitation of traditional antibiotics [100].

Clinical studies demonstrate that C16G2 significantly reduces *S. mutans* levels, thereby lowering caries risk while maintaining microbiome balance. Importantly, after 24 h of treatment, shifts in the microbial community were noted: bacterial species dependent on or interacting with *S. mutans* declined markedly, while natural competitors—such as health-associated *Streptococci*—became dominant. This illustrates C16G2’s potential to modulate oral microbiome structure favorably, highlighting its therapeutic promise [100,101].

Broader antimicrobial stewardship efforts in dentistry increasingly emphasize such targeted approaches. The stewardship involves not just reducing antibiotic use but also precisely modulating the oral microbiota using personalized therapies and novel technologies to improve treatment outcomes and curb resistance development. Their systematic review underscores advanced molecular diagnostics and biotechnological innovations—including CRISPR-Cas systems, photodynamic therapy, probiotics, and targeted peptides like C16G2—as key strategies to minimize broad-spectrum antibiotic reliance and combat resistance emergence effectively [102].

Together, these cutting-edge, targeted microbiome modulation strategies hold great promise for future oral healthcare by efficiently controlling pathogens while preserving beneficial microbes, thus reducing antibiotic resistance and adverse side effects. Continued research and clinical translation of these approaches are essential for sustainable and effective management of oral infections.

### 6.3. Development of Resistance-Informed Clinical Guidelines in Dental Practice

The authors emphasize the need for clinical guidelines in dentistry to be explicitly tailored to the unique characteristics of the oral microbiota, which represents a complex ecosystem crucial for maintaining oral health [100,103]. They advocate for the development of dental practice guidelines grounded on several key pillars. Firstly, the guidelines should prioritize personalized therapy by incorporating recommendations for molecular diagnostic tools that can identify specific pathogens and resistance genes within the oral microbiome. This approach enables the selection of targeted antibiotic therapies, thereby minimizing the use of unnecessary or broad-spectrum antibiotics. Secondly, clinical protocols must include standardized methods for assessing the risk of antimicrobial resistance (AMR) development in individual patients, taking into account factors such as prior antibiotic use, immune status, and specific clinical conditions like periodontitis or implant-related procedures. Thirdly, the guidelines promote the adoption of alternative, non-antibiotic strategies for microbiota modulation, including the use of probiotics, targeted antimicrobial peptides, and photodynamic therapy, all of which help preserve the balance of the oral ecosystem by reducing antibiotic burden. Additionally, the authors highlight the importance of ongoing education for dental professionals on antimicrobial stewardship (AMS) principles, as well as the need for oral-specific AMR surveillance using metagenomic and molecular methods to monitor microbial shifts and detect resistant strains early. They conclude that the development of clinical dental guidelines addressing the complexity of the oral microbiome and the escalating challenge of antibiotic resistance is fundamental for preserving antibiotic efficacy and ensuring patient safety. Such guidelines foster rational, precise, and sustainable management of oral infections, thereby reducing the risk of resistance emergence and dissemination [103].

To further support rational antibiotic prescribing, a mobile application has been developed to assist dentists in making evidence-based decisions, thereby reducing the overuse and unnecessary administration of antibiotics. The application leverages clinical guidelines informed by oral microbiota data and resistance profiles to facilitate accurate diagnosis and the selection of targeted therapies. Dentists input patient symptoms, clinical findings, and preliminary diagnostic results, after which built-in algorithms analyze the likelihood of specific bacterial infections and their resistance patterns. Based on this analysis, the application recommends the most appropriate antibiotic or alternative therapies, such as local treatments or probiotics, while considering the presence of resistance genes and the need for targeted modulation of the microbiota. The application also provides educational content on antibiotic stewardship, the risks of overprescription, and best practices for guideline-based treatment. Furthermore, it enables the monitoring of treatment efficacy and offers feedback for therapy adjustments in cases of poor response or emerging resistance. By facilitating personalized, evidence-based decision-making, the application aims to protect the oral microbiome, curb the development of antibiotic resistance, and promote sustainable therapeutic approaches in dental practice [60,104,105,106,107,108,109].

## Figures and Tables

**Table 1:** Summary of key mechanisms, drivers, and strategies for combating antibiotic resistance in the oral microbiota.

Aspect	Details/Examples	Clinical Implications/Notes	Ref
Major resistance mechanisms	Efflux pumps (e.g., in *Porphyromonas gingivalis*) Enzymatic inactivation (e.g., β-lactamases) Target modification (e.g., erm genes for macrolide resistance) Biofilm formation (e.g., *Candida albicans*)	Reduced efficacy of antibiotics and antifungals Chronic and recurrent infections Need for alternative therapies	[50,51]
Drivers of AMR	Overuse/misuse of antibiotics in dental practice Exposure to antiseptics, biocides, heavy metals Horizontal gene transfer within biofilms	Increased prevalence of multidrug-resistant strains Co-selection of resistance genes	[32]
AMR-associated genes	*tetM* (tetracycline resistance) *ermB*, *mefA/E* (macrolide resistance) *blaZ*, *cfxA* (β-lactam resistance)	Detected in both healthy and diseased individuals High abundance in supragingival biofilms and saliva	[52]
Affected pathogens	*Porphyromonas gingivalis* *Prevotella* spp. *Fusobacterium nucleatum* *Aggregatibacter actinomycetemcomitans* *Candida* spp.	Resistance to amoxicillin, clindamycin, metronidazole, tetracycline, erythromycin, and others	[53]
Alternative therapies	Antimicrobial photodynamic therapy (aPDT) Antimicrobial peptides (AMPs) Probiotics and prebiotics Targeted delivery systems (e.g., nanoparticles, microspheres) Precision-guided peptides (e.g., C16G2)	Reduce reliance on broad-spectrum antibiotics Target pathogens with minimal disruption to microbiota	[54]
Stewardship and surveillance	Education and training of dental professionals Implementation of clinical guidelines Use of diagnostic tools (molecular, metagenomic) Oral-specific AMR surveillance programs	Promotes rational prescribing Enables early detection and targeted interventions	[55]

## Data Availability

All data and materials are available upon request.
